# Psychometric qualities of the patient rated Wrist/Hand evaluation (PRWHE) in dutch primary care patients with wrist complaints

**DOI:** 10.1186/s12875-022-01885-7

**Published:** 2022-11-04

**Authors:** Britt van Gorp, Patrick Krastman, Gerald Kraan, Nina M.C. Mathijssen, Sita M.A. Bierma-Zeinstra, Jos Runhaar

**Affiliations:** 1 Department of General Practice, Erasmus MC University Medical Center Rotterdam, PO-box 2040, 3000 CA Rotterdam, the Netherlands; 2 Reinier de Graaf Groep, Department of Orthopaedic Surgery, Delft, the Netherlands; 3 Department of Orthopedics & Sports Medicine, Erasmus MC University Medical Center, Rotterdam, the Netherlands

**Keywords:** Psychometrics, Questionnaire, Primary care, Wrist injuries

## Abstract

**Background:**

Knowledge on the course, disability and functionality of wrist complaints is still compendious in primary care guidelines, despite the high prevalence in primary care. Valid questionnaires can facilitate the monitoring of patients in primary care and research initiatives. In this study, we aimed to study the psychometric qualities of the Dutch version of the Patient Rated Wrist/Hand Evaluation (PRWHE-DLV) among adults with (sub)acute wrist complaints in primary care.

**Methods:**

An observational cohort of 35 adults with (sub)acute wrist complaints in Dutch primary care was established. The content validity of the PRWHE-DLV was validated by assessing the floor and ceiling effects at baseline (T0). Reproducibility was assessed by the test-retest reliability between T0 and T1 (2–5 days after T0), using the Intra-class Correlation Coefficient. The construct validity was assessed based on the correlation between the PRWHE-DLV and the Quick-DASH, Physical Component Score (SF-12), VAS-function, Physical Functioning (SF-12), VAS-pain and Bodily Pain (SF-12) at T0. Responsiveness was defined as the ability of the PRWHE-DLV to measure change 3 weeks after T0 (internal) and the relation of these changes to clinically important outcomes (external).

**Results:**

Psychometric qualities of the PRWHE-DLV demonstrated high content validity with no floor or ceiling effects, excellent reliability (Intra-class correlation coefficient = 0.90; 95% CI 0.80–0.95), high construct validity with the validated Quick-DASH and VAS score (r = 0.85 with Quick-DASH, r = 0.75 with VAS-function and r = 0.78 with VAS-pain) and high responsiveness.

**Conclusion:**

The PRWHE-DLV provided reliable and adequate information for primary care clinical practice.

## Background

An estimated 34% of patients with hand and/or wrist complaints visit a general practitioner (GP) for medical help [1]. History taking is fundamental to understand the patient’s problem, for the diagnosis and to determine the best treatment strategy. For this, a patient centred approach using a standardized Patient Reported Outcome Measure (PROM) is recommended. Especially when physical examination cannot take place or is inconclusive, questionnaires can provide important information regarding patient status [2]. This may have beneficial effects on the daily activities, concerning the physical, functional and mental state of the patient, leading to less (healthcare) costs and a better productivity [3, 4].

The Patient Rated Wrist Evaluation (PRWE) questionnaire was developed in 1996 [5]. Two years later it was extended to the Patient Rated Wrist/Hand Evaluation (PRWHE) [6]. Previous studies of the original English version of the PRWHE in secondary care have shown strong psychometric qualities and refer to the PRWHE as a reliable, valid and responsive instrument [2, 6–15].

PRWHE has been translated and validated in several countries [16–26]. In 2008, the Dutch version of the PRWHE became available, the PRWHE Dutch Language Version (PRWHE-DLV) [27]. Despite the high prevalence of wrist complaints in Dutch primary care, the psychometric qualities of the PRWHE-DLV are still not sufficiently examined [1]. The only two studies on the psychometric qualities of the PRWHE-DLV found a high internal consistency and test-retest reliability [28] and is an adequate model fit among patients in hospital care [29].

Due to the large number of wrist patients in primary care and the distinctly different patient characteristics compared to secondary/tertiary care, it is of great importance that the psychometric qualities of the PRWHE-DLV are also evaluated in primary care. The aim of this study was to examine the validity (content and hypothesis testing for construct validity), reliability (test-retest reliability and measurement error) and internal and external responsiveness of the PRWHE-DLV among patients with (sub)acute wrist complaints in the Dutch primary care.

## Methods

The study had a prospective cohort design. The study protocol was assessed and approved by the Medical Ethical Committee ZuidWest Holland (16–093), and was in accordance with the ethical standards of the responsible committee on human experimentation and with the Helsinki Declaration of 1975, as revised in 1983. Target sample size of the study was set at 50 patients, as indicated in literature [16–26, 30].

The study followed the COSMIN guidelines to determine which psychometric qualities of the PRWHE-DLV questionnaire were relevant to evaluate [30] and used the STROBE checklist for the study reporting. The PRWHE-DLV is based on a formative model, which was important to take into account when choosing the right psychometric qualities [31]. Measuring internal consistency and structural validity were not considered relevant, as this study did not have the intention to change the actual questionnaire and previous research showed that the PRWHE-DLV measures an unidimensional trait [29]. For the same reason, other items of content validity aimed to change the content of the questionnaire were omitted.

Since a gold standard for (sub)acute wrist complaints is currently unavailable, criterion validity of the PRWHE-DLV was omitted. While taking every psychometric quality into account, the current study aimed to assess content validity, construct validity, reproducibility (test-retest reliability) and construct responsiveness.

Patients were recruited from June 2016 to April 2018 through GPs and by media. A total of 60 GPs in the Netherlands were contacted and requested to inform (sub)acute wrist patients (defined as those patients with complaints less than three months) about this study. Inclusion and exclusion criteria are presented in Table 1.

**Table 1:** Predetermined inclusion and exclusion criteria

Inclusion criteria 1. (Sub)acute wrist pain (traumatic, non-traumatic) 2. Age of 18 year or older 3. Wrist pain located distal of the ulna and radius and proximal of the phalanges 4. Duration of the wrist pain less than 3 months	Exclusion criteria 1. Rheumatic diseases, (poly)arthritis, diabetes or neurological diseases 2. Duration of the wrist pain longer than 3 months 3. Carpal Tunnel Syndrome 4. Pregnancy 5. Patients with infection of their hand 6. Patients with neurovascular pathology 7. Previously trauma of the affected wrist 8. Not capable of the Dutch language

After identification by the GP, an informed consent form had to be signed by the patient. Next, information including the date of inclusion, date of birth, sex, diagnose, patient’s email and phone number was send to the research team. Further information about the study was provided to the patient by the research team and the diagnosis and inclusion/exclusion criteria were checked by phone.

Patients were also recruited through Instagram, Facebook, Twitter and a newspaper advertisement. After registration by email, patients were contacted by telephone within two days. Study information was provided and inclusion and exclusion criteria were checked. All participants provided informed consent prior to any measurements.

The first assessment of patients through an online PROM (T0) was at the day of contacting the patient and used for assessing the validity of the total PRWHE-DLV scores and subscales; T1 was two to five days after T0, and T2 was three weeks after T0.

The online PROM used was composed of six different questionnaires, containing the PRWHE-DLV and five questionnaires for comparison: Quick-Disabilities of the Arm, Shoulder and Hand questionnaire (Quick-DASH); Visual Analog Scale-pain (VAS-pain); Visual Analog Scale-function (VAS-function); Short Form Health Survey (SF-12); and Global Perceived Effect (GPE).

### Psychometric qualities

The content validity was validated by assessing the floor and ceiling effects of the PRWHE-DLV. A limited content validity was deemed present if more than 15% of the patients achieved the lowest (0 to 5) or highest possible score (95 to 100) [32, 33].

To assess whether the PRWHE-DLV produces reproducible, consistent results on repeated administration moments, we tested the test-retest reliability between T0 and T1 [33].

The construct validity was assessed based on the correlation between the PRWHE-DLV and the Quick-DASH, Physical Component Score (SF-12), VAS-function, Physical Functioning (SF-12), VAS-pain and Bodily Pain (SF-12).

Responsiveness was defined as the ability of the PRWHE-DLV to detect clinically important changes over time [33]. In this study, it was hypothesized that the complaints and symptoms of the included patients would change in about three weeks. This time frame was chosen to be long enough to show clinical changes and at the same time short enough to distinguish between (sub)acute and chronic conditions. The Effect Size (ES) and Standardized Response Mean (SRM) were calculated [34]. Comparing the change in PRWHE-DLV with the dichotomized GPE score (‘better’ and ‘much better’ versus the rest) would show the ability to detect (clinically important) changes [34]. Finally, the PRWHE-DLV should be able to differentiate clinically important change from measurement error [34]. Therefore, the Minimal Detectable Change (MDC) to the Minimal important Change (MIC) were calculated [35].

### Missing data

Up to one blank item on a subscale of the PRWHE-DLV was replaced by the mean score of the subscale, according to the directive [36]. To minimize the loss of data, pairwise deletion was used for handling missing values between different analysis and list wise deletion within an analysis for optimization of the outcomes.

### Statistical analysis

Descriptive statistics were used to calculate patients’ characteristics at baseline and were reported as median (Inter Quartile Range; IQR) or mean (Standard Deviation; SD), depending on distribution. Statistically significance was set at a critical value of P < 0.05 (two-tailed). Content validity, floor and ceiling effects were evaluated through Frequencies. Pearson’s rank correlation was used in case of Gaussian distribution, while Spearman rank correlation was used in case of non-Gaussian distribution. Coefficients (r) were used to analyse the construct validity between the (sub-)questionnaires (0 < r < 0.19: very weak; 0.20 < r < 0.39: weak; 0.40 < r < 0.59 moderate; 0.60 < r < 0.79: strong; 0.80 < r < 1.0 very strong.

Test-retest reliability was assessed by determining the Intra-class Correlation Coefficient (ICC two-way mixed single measures[37]) for occasions T0 and T1, after excluding patients with meaningful changes on the GPE (patient who scored ‘much better or worse’ or ‘a lot better or worse’). Excellent reliability was defined as ICC > 0.75, moderate reliability for ICC between 0.40 and 0.75 and a poor reliability for ICC below 0.40 [33, 37].

The standard error of measurement (SEM; SD√(1 – R)) was calculated to define the statistically reliable change. Single measurement was used for test-retest reliability coefficient (R) and the change between T0 and T1 with no meaningful GPE changes (SD) [38]. Responsiveness has been analysed by calculating the effect size (ES; (T2-T0)/SD_T0_) and the standardized response mean (SRM; T2-T0/SD_change_). ). Outcomes were interpreted as strong responsive (> 0.80), moderate (0.50–0.80) or small (0.20–0.50) responsive [34, 39].

This study compared the change in PRWHE-DLV score after three weeks with the dichotomized GPE score for responsiveness with an independent sample T-test or the Mann-Whitney u test - depending on the distribution. Spearman’s or Pearson correlation coefficient was calculated to correlate the changes in PRWHE-DLV after three weeks to the GPE-score. MIC was assessed through defining the mean change score of the PRWHE-DLV for the patient who reported ‘much better’ on the GPE and for the patients who reported ‘much worse’. These outcomes were then subtract to the mean change score of the PRWHE-DLV for patients who reported ‘slightly better’, ‘the same’, ‘slightly worse’ assuming they had no meaningful changes. MDC was calculated through the SEM (MDC = 1.96 x √2 x SEM).

## Results

**Fig. 1:**
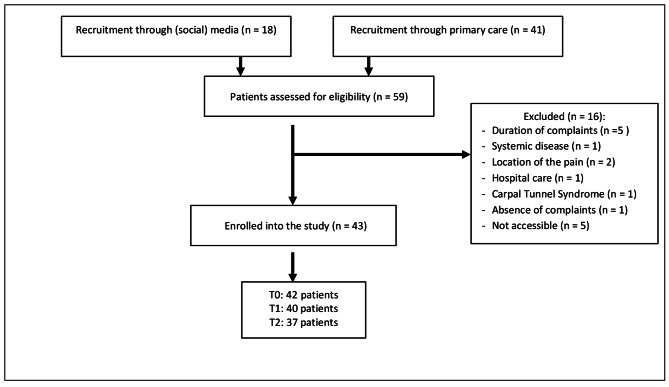
Flowchart showing patient recruitment and follow-up

Of the 60 contacted GPs, 28 GPs wanted to participate. A total of 43 patients (27 women) with a mean age of 44.7 years (SD 17.5) fulfilled the eligibility criteria and were enrolled into the study. Of these, 35 returned all assessments (T0, T1 and T2). Patient recruitment source, reasons for exclusion and follow-up are shown in Fig. 1. The most common diagnosis was overuse/non-specific complaints (n = 9).

At follow-up, the median duration between baseline and T1 was 47.5 h (n = 40; IQR = 11) and 21 days (n = 36; IQR = 1) at T2.

### Content validation

None of the 97 completed questionnaires had the best score (95–100 points) and 12.7% scored between zero and five.

### Reproducibility (test-retest reliability)

In total, 35 patients returned completed questionnaires for analysis on T0 and T1. Six patients reported a meaningful change on the GPE at the second assessment and were thus excluded for this analysis. Test–retest reliability for the PRWHE-DLV total (n = 29; ICC = 0.90; 95% CI 0.80–0.95), PRWHE-DLV function (n = 29; ICC = 0.90; 95% CI 0.80–0.95) and PRWHE-DLV pain (n = 32; ICC = 0.86; 95% CI 0.74–0.93) was excellent. SEM (n = 29) was 3 (9.48 * √0.10).

### Construct validation

Correlations for construct validation are presented in Table 2. In general there was a moderate to strong correlation of the PRWHE-DLV with the different questionnaires for comparison, apart from the correlation between the total PRWHE score and PCS, and between the PRWHE Function score and the Physical Functioning (SF-12); both weak (r = -0.37).

**Table 2:** Correlation coefficients (r) determined at T0 when comparing PRWHE-DLV (sub)scales to the Quick-DASH, PCS, VAS-pain, Bodily pain, VAS-function an Physical function

PRWHE-Score*		Quick-DASH *(n = 33)*	PCS^†^ *(n = 36)*	VAS-P^‡^ *(n = 39)*	BP ^¶^ *(n = 40)*	VAS-F ^||^ *(n = 36)*	PF ^§^ *(n = 36)*
Total	r	0.85	-0.37				
Pain	r			0.78	-0.50		
Function	r					0.75	-0.37

*PRWHE: Patient Rated Wrist Hand Evaluation; ^†^PCS: Physical Component Score; ^‡^VAS-P: Visual Analog Scale for pain; ^¶^BP: Bodily Pain; ^||^ VAS-F: Visual Analog Scale for function; ^§^PF: Physical Function.

### Construct responsiveness

Thirty patients completed the PRWHE-DLV and GPE score at T2. ES and SRM were calculated to be strong responsive (ES = -1.05, SRM = -0.96). The PCC showed a significantly linear correlation between the change of the PRWHE-DLV score during follow-up and the GPE score (Pearson correlation coefficient r = -0.73; p < 0.001). Patients with no meaningful changes (n = 13) scored a decrease in mean of 9.73 (95%CI = -17.95 to -1.51) and patients who defined their complaints as much better (n = 11) scored − 22.86 (95%CI = -31.98 to -13.75). This resulted in a MIC of -13.13. MDC was calculated for 29 patients to be 8.32.

## Discussion

### Summary

In this prospective cohort study, content validity, test-retest reliability, and internal and external responsiveness of the PRWHE-DLV questionnaire in assessing (sub)acute complaints of pain and/or function loss of the wrist among adults in the Dutch primary care setting were evaluated. This study showed that the PRWHE-DLV is a valid, reliable and responsive instrument for patients with (sub)acute wrist complaints in Dutch primary care.

### Strengths and limitations

Research into the psychometric qualities of the PRWHE-DLV has never been performed before for patients with (sub)acute wrist complaints in primary care. The current study provided an elaborate overview of the psychometric qualities of this PROM for this specific population. Compared to existing literature, the current study uses a broad array of psychometric qualities. No floor and ceiling effects were found, which confirms the positive interpretability of the items in the questionnaires and avoids unreliable answers and missing values [40].

However, there are some limitations to this study. Firstly, the small sample size, possible selection of well-motivated patients, and the fact that ± 30% of patients were recruitment outside of primary care should be taken into account when interpreting the results of this study. However, the mean age, male to female ratio, and the wide spectrum of disorders of the cohort were comparable to the Dutch population with hand and wrist disorders [1]. Moreover, also all patients recruited through (social) media had a symptom duration < 3 months and had all pre-specified conditions excluded. Therefore, we assumed these patients to be reflective of primary care patients. Secondly, the used pairwise deletion may result in an inconsistency of the sample size. Nevertheless, the pairwise deletion was only used for handling missing values between different analysis and list wise deletion within an analysis for optimization of the outcomes. Finally, not all aspects of content validity were assessed in the current study, since we did not aim to change the outline of the questionnaire. The evaluation of the floor and ceiling effects doesn’t guarantee that no important items are missing in the current version of the PRWHE-DLV. Therefore, more research, including qualitative methods, is required to obtain a complete assessment of the content validity of the PRWHE-DLV.

### Comparison with existing literature

The main results of this study are consistent with the findings of previous studies. Our reported ICCs (type 3,1) are similar in direction and size to those reported in the original version[6] and in the adapted versions for other languages [7, 16, 19–26, 28]. Nevertheless, it should be noted that not all studies used the same ICC type and some of the studies did not report the ICC type. This could explain the minimal variety between the results of previously published studies. Many of the previous studies concerned other populations; mainly patients who had conditions like Distal Radius fractures and osteoarthritis recruited in hospital care.

Previous studies also reported strong correlations with the (Quick-)DASH, VAS-pain and VAS-function [6, 10, 16–23, 28, 36, 41, 42]. MacDermid et al. found a correlation with PCS of r = 0.63 and a correlation with BP of r = 0.72, while this study and other studies showed a lower correlation for these scales [6]. This could be explained by the dominance of lower extremity items in the SF-12/SF-36, which are less specific for hand and/or wrist complaints in contrast to the Quick-DASH.

A strong responsive ES and SRM were found in this study, although most studies reported a higher ES and SRM, varying between 0.84 and 3.46 for ES[2, 6, 7, 10, 15, 18, 20, 22, 23] and between 0.89 and 1.94 for SRM [2, 7, 10, 18–20, 22, 23]. This might be related to the population criteria, treatments and follow-up assessment times. This variation in relation to the population criteria, treatments and follow-up assessment times also causes dissimilarity for outcomes like the MIC (ranges between 10.2 and 24.0) and MDC (varying between 4.4 and 12.5) [7, 22, 23, 26].

### Implication for research and/or practice

This study provided evidence that the PRWHE-DLV can be used to assess adults with (sub)acute wrist complaints in Dutch primary care. It showed an adequate validity, reliability and responsiveness of the PRWHE-DLV. A patient centred approach, such as the PRWHE-DLV, supports public accountability of healthcare and gives a reliable overview about patients’ complaints [43, 44]. In addition, by quantifying subjective complaints and disabilities the PROM prevents observer bias and enables the GP to monitor the symptoms in time [43, 44]. These quantifying outcomes can then be used for further research. The notable positive psychometric qualities in combination with the positive effect of a PROM emphasized the high relevance of the PRWHE-DLV to be used in the future. Given the similarities in primary care settings, current results are also encouraging for using the PRWHE to assess patients with wrist complaints in primary care in other Western countries.

## Conclusion

Based on the measures obtained in the current study, the PRWHE-DLV is a valid, reliable and responsive instrument for the assessment of (sub)acute complaints of pain and/or function loss of the wrist patients with (sub)acute wrist complaints in primary care.

## Data Availability

The datasets generated and analysed during the current study are not publicly available due to lack of patients’ consent for this, but are available from the corresponding author on reasonable request.

## Abbreviations

ES: Effect Size
GP: General Practitioner
GPE: Global Perceived Effect
ICC: Intra-class Correlation Coefficient
IQR: Inter Quartile Range
MDC: Minimal Detectable Change
MIC: Minimal Important Change
PCC: Pearson correlation coefficient
PROM: Patient Reported Outcome Measure
PRWHE: Patient Rated Wrist/Hand Evaluation
PRWHE-DLV: Patient Rated Wrist/Hand Evaluation – Dutch language Version
Quick-DASH: Quick-Disabilities of the Arm, Shoulder and Hand questionnaire
SD: Standard Deviation
SEM: Standard Error of Measurement
SF: Short Form Health Survey
SRM: Standardized Response Mean
VAS: Visual Analog Scale
